# 2,2′-{[2-(2-Chloro­phen­yl)-4-methyl­imidazolidine-1,3-di­yl]bis­(methyl­ene)}diphenol

**DOI:** 10.1107/S1600536813017923

**Published:** 2013-07-10

**Authors:** Augusto Rivera, Lorena Cárdenas, Jaime Ríos-Motta, Monika Kučeraková, Michal Dušek

**Affiliations:** a Universidad Nacional de Colombia, Sede Bogotá, Facultad de Ciencias, Departamento de Química, Cra 30 No.45-03, Bogotá, Código Postal 111321, Colombia; b Institute of Physics ASCR, v.v.i., Na Slovance 2, 182 21 Praha 8, Czech Republic

## Abstract

In the title compound, C_24_H_25_ClN_2_O_2_, the 2-hy­droxy­benzyl substituents and the 2-chloro­phenyl group occupy the sterically preferred equatorial positions, whereas the methyl group occupies the axial position. The imidazolidine ring adopts an envelope conformation with one of the N atoms adjacent to the methylene group as the flap. The chloro­phenyl substit­uent approaches a nearly perpendicular orientation relative to the mean plane of the imidazolidine ring, making a dihedral angle of 73.44 (12)° and the Cl atom is almost coplanar with the C atom bearing the chloro­phenyl substituent [Cl—C—C—C torsion angle = 1.1 (3)°]. The hy­droxy­benzyl groups make dihedral angles of 71.23 (15) and 69.13 (19)° with the mean plane of the heterocyclic ring. The dihedral angle between the two hy­droxy­benzyl groups is 69.61 (12)°. The mol­ecular structure features two intra­molecular O—H⋯N hydrogen bonds with graph-set motif *S*(6) between the phenolic hydroxyl groups and N atoms.

## Related literature

For related structures, see: Rivera *et al.* (2012*a* ▶,*b* ▶). For the synthesis of the title compound, see: Rivera *et al.* (2013 ▶). For bond-length data, see: Allen *et al.* (1987 ▶). For hydrogen-bond graph-set nomenclature, see: Bernstein *et al.* (1995 ▶).
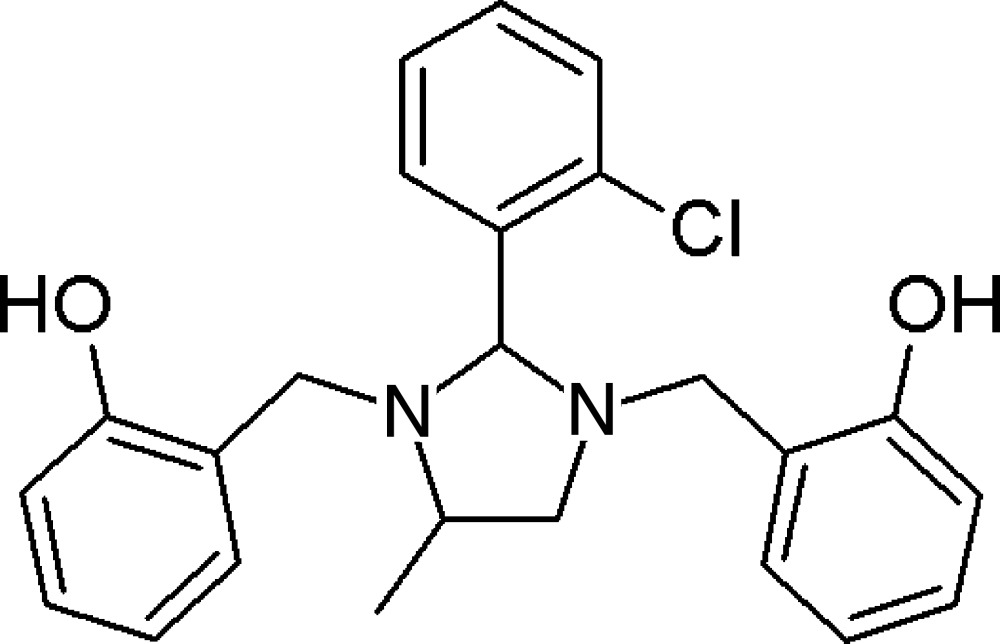


## Experimental

### 

#### Crystal data

C_24_H_25_ClN_2_O_2_
*M* *_r_* = 408.9
Monoclinic, 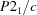

*a* = 7.0281 (2) Å
*b* = 9.7903 (3) Å
*c* = 30.3813 (6) Å
β = 94.168 (2)°
*V* = 2084.92 (10) Å^3^
*Z* = 4
Mo *K*α radiation
μ = 0.21 mm^−1^
*T* = 120 K
0.24 × 0.15 × 0.08 mm

#### Data collection

Agilent Xcalibur (Atlas, Gemini ultra) diffractometer
Absorption correction: multi-scan (*CrysAlis PRO*; Agilent, 2010 ▶) *T*_min_ = 0.392, *T*_max_ = 1
25057 measured reflections
3732 independent reflections
3164 reflections with *I* > 3σ(*I*)
*R*_int_ = 0.034

#### Refinement

*R*[*F* ^2^ > 2σ(*F* ^2^)] = 0.057
*wR*(*F* ^2^) = 0.081
*S* = 2.66
3732 reflections
268 parameters
1 restraint
H-atom parameters constrained
Δρ_max_ = 1.29 e Å^−3^
Δρ_min_ = −0.33 e Å^−3^

### 

Data collection: *CrysAlis PRO* (Agilent, 2010 ▶); cell refinement: *CrysAlis PRO*; data reduction: *CrysAlis PRO*; program(s) used to solve structure: *SUPERFLIP* (Palatinus & Chapuis 2007 ▶); program(s) used to refine structure: *JANA2006* (Petříček *et al.* 2006 ▶); molecular graphics: *DIAMOND* (Brandenburg & Putz, 2005 ▶); software used to prepare material for publication: *JANA2006*.

## Supplementary Material

Crystal structure: contains datablock(s) global, I. DOI: 10.1107/S1600536813017923/bx2443sup1.cif

Structure factors: contains datablock(s) I. DOI: 10.1107/S1600536813017923/bx2443Isup2.hkl

Supplementary material file. DOI: 10.1107/S1600536813017923/bx2443Isup3.cml

Additional supplementary materials:  crystallographic information; 3D view; checkCIF report

## Figures and Tables

**Table 1:** Hydrogen-bond geometry (Å, °)

*D*—H⋯*A*	*D*—H	H⋯*A*	*D*⋯*A*	*D*—H⋯*A*
O1—H1⋯N2	1.01 (3)	1.79 (3)	2.721 (3)	152 (3)
O2—H2⋯N1	1.01 (3)	1.79 (3)	2.723 (3)	152 (3)

**Fig. 1.**
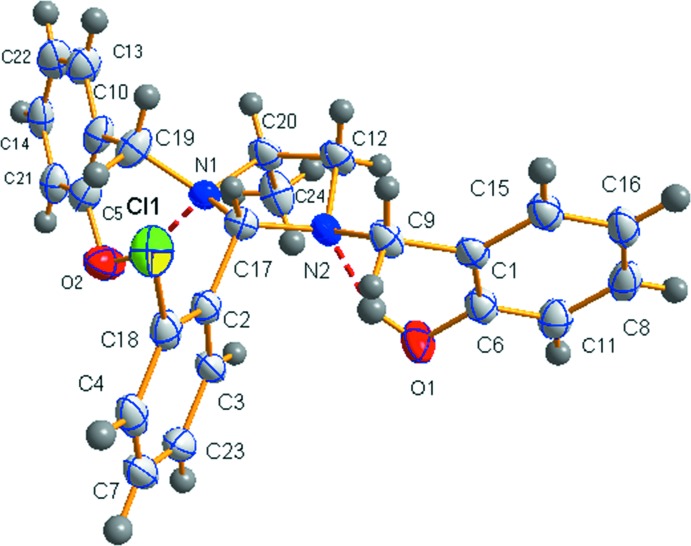
A perspective view of the title compound. Displacement ellipsoids are drawn at the 50% probability level and H atoms are shown as small spheres of arbitrary radii. Hydrogen bonds are drawn as dashed lines.

## supplementary crystallographic information

### Comment

As a continuation of systematic studies into the synthesis, characterization and structural properties of substituted Mannich bases, crystals of the title compound were isolated and characterized crystallographically.

In the title compound (Fig. 1), all values of the geometric parameters are normal (Allen *et al.*, 1987). The largest difference maximum 1.2 e^-^ A^-3^ coincides with the hydrogen H1—C13 and it could be explained by an atom with the scattering power of 2.2 H atoms. We tried to describe this maximum with an O—H group disordered between C5 (occupancy 0.85) and C13 (occupancy 0.15). The difference maximum decreased to ~0.5 e^-^ A^-3^, which unfortunately still dominates the difference electron density map and remains at the position of the partially occupied (0.85) H1—C13. Because this disorder complicates the structure description and at the same time does not fully explain the difference maximum, we used for the final CIF the structure model without disorder.

The distances within the imidazolidine ring of the title compund are very similar to those found in related strctures (Rivera *et al.*, 2012*a*,*b*). However, the observed N1—C20 bond length [1.498 (3) Å] is shorter in relation to the value observed in related structure [1.513 (2) Å] (Rivera *et al.*, 2012*a*). The central imidazolidine core bearing a chlorophenyl, a methyl as well as two *ortho*-hydroxybenzyl groups as substituents, while the orientation of the *ortho-*hydroxybenzyl substituents on both sides of the imidazolidine ring and the chlorinated phenyl group are the sterically preferred equatorial positions and only the methyl group adopts a axial orientation. The mean plane of the imidazolidine ring defined by C17, N1, C20 makes a dihedral angle of 73.44 (12)° with the chlorophenyl substituent and the chlorine atom is almost coplanar with the C atom bearing the chlorophenyl substituent [Cl1—C18—C2—C17 torsion angle == 1.05 (31)°]. The hydroxybenzyl groups makes an angle of 71.23 (15)° and 69.13 (19)° with the mean plane of heterocyclic ring. The dihedral angle between the two hydroxybenzyl groups is 69.61 (12)°.

The crystal structure of the title confirms the presence of two O—H···N(1,3-imidazolidine) hydrogen bond with graph-set motif S(6) (Bernstein *et al.* 1995) (Table 1). The N···O distances [N1···O2, 2.723 (3) and N2···O1, 2.721 (3) Å] is longer in comparison with the values observed in related structure (Rivera, *et al.* 2012*b*), showing a slightly decrease in hydrogen-bonding strength.

### Experimental

For the originally reported synthesis, see: Rivera *et al.* (2013). Single crystals of the title compound (I) were grown from ethanol by recrystallization

### Refinement

The hydroxyl hydrogen atoms were found in difference Fourier maps and their coordinates were refined with a distance restraint d(O—H) = 1.012 Å with σ 0.01. All other H atoms atoms were kept in the geometrically correct positions with C—H distance 0.96 A. The isotropic atomic displacement parameters of hydrogen atoms were evaluated as 1.2×*U*_eq_ of the parent atom.

### Crystal data

**Table:**

C_24_H_25_ClN_2_O_2_	*F*(000) = 864
*M**_r_* = 408.9	*D*_x_ = 1.302 Mg m^−^^3^
Monoclinic, *P*2_1_/*c*	Mo *K*α radiation, λ = 0.71069 Å
Hall symbol: -P 2ycb	Cell parameters from 10626 reflections
*a* = 7.0281 (2) Å	θ = 2.9–67.0°
*b* = 9.7903 (3) Å	µ = 0.21 mm^−^^1^
*c* = 30.3813 (6) Å	*T* = 120 K
β = 94.168 (2)°	Polygon shape, white
*V* = 2084.92 (10) Å^3^	0.24 × 0.15 × 0.08 mm
*Z* = 4	

### Data collection

**Table:**

Agilent Xcalibur (Atlas, Gemini ultra) diffractometer	3732 independent reflections
Radiation source: Enhance Ultra (Cu) X-ray Source	3164 reflections with *I* > 3σ(*I*)
Mirror monochromator	*R*_int_ = 0.034
Detector resolution: 10.3784 pixels mm^-1^	θ_max_ = 25.1°, θ_min_ = 1.3°
ω scans	*h* = −8→8
Absorption correction: multi-scan (*CrysAlis PRO*; Agilent, 2010)	*k* = −11→11
*T*_min_ = 0.392, *T*_max_ = 1	*l* = −36→31
25057 measured reflections	

### Refinement

**Table:**

Refinement on *F*	94 constraints
*R*[*F*^2^ > 2σ(*F*^2^)] = 0.057	H-atom parameters constrained
*wR*(*F*^2^) = 0.081	Weighting scheme based on measured s.u.'s *w* = 1/(σ^2^(*F*) + 0.0004*F*^2^)
*S* = 2.66	(Δ/σ)_max_ = 0.024
3732 reflections	Δρ_max_ = 1.29 e Å^−^^3^
268 parameters	Δρ_min_ = −0.33 e Å^−^^3^
1 restraint	

### Special details

**Table:**

Experimental. (CrysAlis PRO; Agilent, 2010) Empirical absorption correction using spherical harmonics, implemented in SCALE3 ABSPACK scaling algorithm.
Refinement. The refinement was carried out against all reflections. The conventional *R*-factor is always based on *F*. The goodness of fit as well as the weighted *R*-factor are based on *F* and *F*^2^ for refinement carried out on *F* and *F*^2^, respectively. The threshold expression is used only for calculating *R*-factors *etc*. and it is not relevant to the choice of reflections for refinement.The program used for refinement, Jana2006, uses the weighting scheme based on the experimental expectations, see _refine_ls_weighting_details, that does not force *S* to be one. Therefore the values of *S* are usually larger than the ones from the *SHELX* program.

### Fractional atomic coordinates and isotropic or equivalent isotropic displacement parameters (Å^2^)

**Table:**

	*x*	*y*	*z*	*U*_iso_*/*U*_eq_	
Cl1	0.47526 (9)	0.37100 (7)	0.066197 (19)	0.0426 (2)	
O1	1.1631 (3)	−0.0315 (2)	0.09734 (7)	0.0521 (7)	
N1	0.6652 (3)	0.10467 (19)	0.17016 (6)	0.0276 (5)	
N2	0.7887 (3)	0.02087 (19)	0.10763 (6)	0.0281 (5)	
O2	0.8058 (3)	0.2490 (2)	0.24193 (6)	0.0533 (7)	
C1	0.9092 (3)	−0.1023 (2)	0.04481 (7)	0.0313 (7)	
C2	0.7791 (3)	0.2711 (2)	0.11711 (7)	0.0303 (6)	
C3	0.9648 (4)	0.2880 (2)	0.13809 (7)	0.0358 (7)	
C4	0.8047 (4)	0.5012 (3)	0.08815 (8)	0.0408 (8)	
C6	1.0958 (4)	−0.1154 (3)	0.06367 (8)	0.0373 (7)	
C5	0.6590 (4)	0.1942 (3)	0.26237 (8)	0.0398 (8)	
C7	0.9840 (4)	0.5144 (3)	0.10888 (8)	0.0439 (8)	
C8	1.1541 (4)	−0.3039 (3)	0.01561 (7)	0.0405 (8)	
C9	0.7795 (3)	0.0089 (2)	0.05926 (7)	0.0327 (7)	
C10	0.5015 (4)	0.1449 (3)	0.23719 (8)	0.0374 (7)	
C11	1.2177 (4)	−0.2147 (3)	0.04910 (8)	0.0420 (8)	
C12	0.7072 (4)	−0.0978 (2)	0.12959 (7)	0.0361 (7)	
C13	0.3618 (4)	0.0796 (3)	0.25849 (9)	0.0468 (9)	
C14	0.5252 (4)	0.1215 (3)	0.32865 (8)	0.0440 (9)	
C15	0.8499 (4)	−0.1925 (3)	0.01111 (7)	0.0375 (7)	
C16	0.9707 (4)	−0.2924 (3)	−0.00355 (8)	0.0415 (8)	
C17	0.6776 (3)	0.1362 (2)	0.12301 (7)	0.0286 (6)	
C18	0.7034 (4)	0.3801 (2)	0.09253 (7)	0.0334 (7)	
C19	0.4922 (4)	0.1602 (3)	0.18752 (8)	0.0410 (8)	
C20	0.6855 (4)	−0.0468 (2)	0.17574 (8)	0.0383 (8)	
C21	0.6697 (4)	0.1850 (3)	0.30818 (8)	0.0436 (8)	
C22	0.3725 (5)	0.0669 (3)	0.30433 (9)	0.0513 (10)	
C23	1.0644 (4)	0.4083 (3)	0.13432 (8)	0.0407 (8)	
C24	0.8613 (5)	−0.0821 (3)	0.20628 (9)	0.0509 (9)	
H1c3	1.021974	0.214303	0.155206	0.043*	
H1c4	0.749472	0.574892	0.070745	0.049*	
H1c7	1.054201	0.597549	0.105794	0.0527*	
H1c8	1.237584	−0.373425	0.005822	0.0486*	
H1c9	0.815069	0.094296	0.046706	0.0392*	
H2c9	0.650655	−0.010354	0.04832	0.0392*	
H1c11	1.345868	−0.22167	0.062155	0.0504*	
H1c12	0.796755	−0.171914	0.130535	0.0433*	
H2c12	0.583691	−0.118423	0.115581	0.0433*	
H1c13	0.254234	0.041637	0.241431	0.0562*	
H1c14	0.531698	0.11563	0.360265	0.0528*	
H1c15	0.722369	−0.185392	−0.002335	0.045*	
H1c16	0.926718	−0.353119	−0.026918	0.0499*	
H1c17	0.555538	0.146235	0.106973	0.0344*	
H1c19	0.382765	0.112164	0.174649	0.0492*	
H2c19	0.480064	0.255055	0.179876	0.0492*	
H1c20	0.579161	−0.087302	0.188978	0.0459*	
H1c21	0.777132	0.222541	0.325396	0.0523*	
H1c22	0.27375	0.020425	0.31863	0.0616*	
H1c23	1.188841	0.418716	0.1492	0.0489*	
H1c24	0.871865	−0.179534	0.209113	0.0611*	
H2c24	0.973392	−0.046657	0.194076	0.0611*	
H3c24	0.848806	−0.042368	0.234811	0.0611*	
H1	1.042 (5)	0.005 (4)	0.1092 (11)	0.0625*	
H2	0.785 (5)	0.212 (4)	0.2108 (10)	0.0639*	

### Atomic displacement parameters (Å^2^)

**Table:**

	*U*^11^	*U*^22^	*U*^33^	*U*^12^	*U*^13^	*U*^23^
Cl1	0.0468 (4)	0.0437 (4)	0.0364 (3)	0.0097 (3)	−0.0034 (2)	0.0023 (2)
O1	0.0435 (11)	0.0589 (13)	0.0526 (11)	−0.0001 (9)	−0.0056 (8)	−0.0247 (9)
N1	0.0304 (9)	0.0255 (10)	0.0272 (9)	−0.0014 (7)	0.0043 (7)	0.0034 (7)
N2	0.0372 (10)	0.0226 (9)	0.0243 (9)	−0.0034 (7)	0.0009 (7)	−0.0007 (7)
O2	0.0551 (12)	0.0583 (13)	0.0464 (11)	−0.0161 (10)	0.0039 (8)	−0.0061 (9)
C1	0.0434 (13)	0.0267 (12)	0.0243 (10)	−0.0071 (9)	0.0052 (8)	0.0018 (8)
C2	0.0372 (12)	0.0283 (12)	0.0259 (10)	−0.0002 (9)	0.0068 (8)	−0.0010 (8)
C3	0.0466 (13)	0.0259 (12)	0.0361 (12)	−0.0080 (10)	0.0104 (10)	−0.0046 (9)
C4	0.0672 (17)	0.0270 (12)	0.0306 (12)	−0.0012 (11)	0.0196 (11)	−0.0022 (9)
C6	0.0465 (14)	0.0359 (13)	0.0295 (11)	−0.0031 (10)	0.0026 (9)	−0.0035 (9)
C5	0.0427 (13)	0.0392 (14)	0.0384 (12)	0.0076 (11)	0.0093 (10)	0.0087 (10)
C7	0.0622 (17)	0.0301 (13)	0.0420 (13)	−0.0130 (12)	0.0209 (11)	−0.0062 (10)
C8	0.0570 (16)	0.0358 (14)	0.0304 (11)	0.0025 (11)	0.0143 (10)	0.0024 (9)
C9	0.0434 (13)	0.0281 (12)	0.0258 (11)	−0.0033 (9)	−0.0021 (9)	0.0003 (8)
C10	0.0397 (13)	0.0395 (14)	0.0337 (12)	0.0115 (10)	0.0073 (9)	0.0046 (9)
C11	0.0479 (15)	0.0433 (15)	0.0349 (12)	0.0027 (12)	0.0047 (10)	0.0007 (10)
C12	0.0491 (14)	0.0249 (12)	0.0344 (12)	−0.0045 (10)	0.0034 (10)	0.0047 (9)
C13	0.0526 (16)	0.0492 (16)	0.0402 (14)	0.0001 (13)	0.0151 (11)	−0.0013 (11)
C14	0.0668 (18)	0.0326 (13)	0.0342 (13)	0.0154 (12)	0.0142 (11)	0.0040 (9)
C15	0.0483 (14)	0.0361 (13)	0.0282 (11)	−0.0084 (11)	0.0036 (9)	−0.0035 (9)
C16	0.0624 (17)	0.0344 (14)	0.0291 (11)	−0.0084 (12)	0.0113 (10)	−0.0072 (9)
C17	0.0296 (11)	0.0293 (12)	0.0266 (10)	−0.0010 (9)	−0.0002 (8)	0.0019 (8)
C18	0.0453 (13)	0.0308 (12)	0.0250 (10)	0.0022 (10)	0.0092 (9)	−0.0022 (8)
C19	0.0355 (12)	0.0549 (16)	0.0329 (12)	0.0113 (11)	0.0047 (9)	0.0066 (10)
C20	0.0508 (14)	0.0277 (12)	0.0370 (12)	−0.0068 (10)	0.0080 (10)	0.0035 (9)
C21	0.0529 (15)	0.0400 (14)	0.0379 (13)	0.0119 (12)	0.0042 (11)	0.0043 (11)
C22	0.0700 (19)	0.0444 (16)	0.0423 (14)	−0.0010 (14)	0.0235 (13)	0.0041 (12)
C23	0.0421 (13)	0.0366 (14)	0.0449 (13)	−0.0096 (11)	0.0121 (10)	−0.0088 (10)
C24	0.0688 (19)	0.0407 (16)	0.0413 (14)	0.0111 (13)	−0.0097 (12)	−0.0010 (11)

### Geometric parameters (Å, º)

**Table:**

O1—C6	1.369 (3)	C9—H1c9	0.96
N1—C17	1.474 (3)	C9—H2c9	0.96
N1—C19	1.465 (3)	C10—C13	1.373 (4)
N1—C20	1.498 (3)	C10—C19	1.513 (3)
N2—C9	1.471 (3)	C11—H1c11	0.96
N2—C12	1.476 (3)	C12—C20	1.507 (3)
N2—C17	1.468 (3)	C12—H1c12	0.96
O2—C5	1.353 (3)	C12—H2c12	0.96
C1—C6	1.398 (3)	C13—C22	1.395 (4)
C1—C9	1.506 (3)	C13—H1c13	0.96
C1—C15	1.392 (3)	C14—C21	1.377 (4)
C2—C3	1.420 (3)	C14—C22	1.367 (4)
C2—C17	1.518 (3)	C14—H1c14	0.96
C2—C18	1.386 (3)	C15—C16	1.389 (4)
C3—C23	1.380 (4)	C15—H1c15	0.96
C3—H1c3	0.96	C16—H1c16	0.96
C4—C7	1.373 (4)	C17—H1c17	0.96
C4—C18	1.394 (4)	C19—H1c19	0.96
C4—H1c4	0.96	C19—H2c19	0.96
C6—C11	1.389 (4)	C20—C24	1.529 (4)
C5—C10	1.386 (3)	C20—H1c20	0.96
C5—C21	1.391 (3)	C21—H1c21	0.96
C7—C23	1.390 (4)	C22—H1c22	0.96
C7—H1c7	0.96	C23—H1c23	0.96
C8—C11	1.390 (4)	C24—H1c24	0.96
C8—C16	1.380 (4)	C24—H2c24	0.96
C8—H1c8	0.96	C24—H3c24	0.96
			
C17—N1—C19	112.41 (17)	H1c12—C12—H2c12	115.07
C17—N1—C20	107.79 (17)	C10—C13—C22	121.6 (3)
C19—N1—C20	113.64 (19)	C10—C13—H1c13	119.2
C9—N2—C12	113.49 (17)	C22—C13—H1c13	119.2
C9—N2—C17	113.18 (17)	C21—C14—C22	120.5 (2)
C12—N2—C17	103.20 (17)	C21—C14—H1c14	119.73
C6—C1—C9	121.1 (2)	C22—C14—H1c14	119.73
C6—C1—C15	117.7 (2)	C1—C15—C16	121.6 (2)
C9—C1—C15	121.1 (2)	C1—C15—H1c15	119.18
C3—C2—C17	118.20 (19)	C16—C15—H1c15	119.18
C3—C2—C18	117.2 (2)	C8—C16—C15	119.8 (2)
C17—C2—C18	124.6 (2)	C8—C16—H1c16	120.12
C2—C3—C23	121.2 (2)	C15—C16—H1c16	120.12
C2—C3—H1c3	119.42	N1—C17—N2	102.62 (16)
C23—C3—H1c3	119.42	N1—C17—C2	110.98 (17)
C7—C4—C18	119.6 (2)	N1—C17—H1c17	113.65
C7—C4—H1c4	120.18	N2—C17—C2	111.48 (18)
C18—C4—H1c4	120.18	N2—C17—H1c17	113.17
O1—C6—C1	120.9 (2)	C2—C17—H1c17	105.14
O1—C6—C11	118.1 (2)	C2—C18—C4	121.8 (2)
C1—C6—C11	121.0 (2)	N1—C19—C10	110.23 (19)
O2—C5—C10	119.3 (2)	N1—C19—H1c19	109.47
O2—C5—C21	119.8 (2)	N1—C19—H2c19	109.47
C10—C5—C21	120.9 (2)	C10—C19—H1c19	109.47
C4—C7—C23	120.5 (2)	C10—C19—H2c19	109.47
C4—C7—H1c7	119.77	H1c19—C19—H2c19	108.7
C23—C7—H1c7	119.76	N1—C20—C12	103.76 (18)
C11—C8—C16	119.9 (2)	N1—C20—C24	111.0 (2)
C11—C8—H1c8	120.06	N1—C20—H1c20	112.71
C16—C8—H1c8	120.06	C12—C20—C24	111.0 (2)
N2—C9—C1	111.43 (18)	C12—C20—H1c20	112.78
N2—C9—H1c9	109.47	C24—C20—H1c20	105.75
N2—C9—H2c9	109.47	C5—C21—C14	119.5 (2)
C1—C9—H1c9	109.47	C5—C21—H1c21	120.23
C1—C9—H2c9	109.47	C14—C21—H1c21	120.23
H1c9—C9—H2c9	107.44	C13—C22—C14	119.2 (3)
C5—C10—C13	118.1 (2)	C13—C22—H1c22	120.39
C5—C10—C19	119.4 (2)	C14—C22—H1c22	120.39
C13—C10—C19	122.4 (2)	C3—C23—C7	119.7 (2)
C6—C11—C8	120.0 (2)	C3—C23—H1c23	120.14
C6—C11—H1c11	120	C7—C23—H1c23	120.14
C8—C11—H1c11	120	C20—C24—H1c24	109.47
N2—C12—C20	103.23 (18)	C20—C24—H2c24	109.47
N2—C12—H1c12	109.47	C20—C24—H3c24	109.47
N2—C12—H2c12	109.47	H1c24—C24—H2c24	109.47
C20—C12—H1c12	109.47	H1c24—C24—H3c24	109.47
C20—C12—H2c12	109.47	H2c24—C24—H3c24	109.47

### Hydrogen-bond geometry (Å, º)

**Table:**

*D*—H···*A*	*D*—H	H···*A*	*D*···*A*	*D*—H···*A*
O1—H1···N2	1.01 (3)	1.79 (3)	2.721 (3)	152 (3)
O2—H2···N1	1.01 (3)	1.79 (3)	2.723 (3)	152 (3)
O1—H1···C9	1.01 (3)	2.31 (3)	2.883 (3)	115 (2)
O2—H2···C19	1.01 (3)	2.19 (4)	2.796 (3)	117 (3)
